# Cyclic Fatigue Resistance of Rotary versus Reciprocating Endodontic Files: A Systematic Review and Meta-Analysis

**DOI:** 10.3390/jcm13030882

**Published:** 2024-02-02

**Authors:** Ana De Pedro-Muñoz, Cristina Rico-Romano, Patricia Sánchez-Llobet, José María Montiel-Company, Jesús Mena-Álvarez

**Affiliations:** 1 Department of Endodontics, Faculty of Dentistry, Alfonso X El Sabio University, 28691 Madrid, Spain; amunodep@uax.es (A.D.P.-M.); cromaric@uax.es (C.R.-R.); aqx181756@uic.es (P.S.-L.); 2 Department of Stomatology, Faculty of Medicine and Dentistry, University of Valencia, 46010 Valencia, Spain; jose.maria.montiel@uv.es

**Keywords:** cyclic fatigue resistance, endodontic instruments, fatigue properties, NiTi alloy, NiTi files, stress fracture, stress resistance

## Abstract

(1) **Background**: The failure of nickel–titanium (NiTi) rotary files is a complication related to endodontic instruments. The aim of this study was to compare the resistance to cyclic fatigue between rotary and reciprocating file systems. (2) **Methods**: Specific PICO: Population (P): artificial root canals; Interventions (I): instrumentation with NiTi rotary and reciprocating files; Comparison (C): rotary versus reciprocating files; Outcome (O): cyclic fatigue resistance. Studies were identified through bibliographic research using electronic databases (Medline, Embase, Scopus, SciELO, and WOS). The studies were combined using a random effects model by the inverse variance method. The effect size was the mean of the time to fracture (TTF) and number of cycles to fracture (NCF). Heterogeneity was assessed using the p value of the Q test for heterogeneity and the I2. (3) **Results**: TTF for rotary files was determined in 474.5 s and 839.1 for reciprocating without statistically significant differences. NCF for rotary systems was determined in 1444.2 and for reciprocating file systems in 4155.9 with statistically significant differences (*p* = 0.035), making reciprocating files more resistant. (4) **Conclusions**: Reciprocating files have better resistance to cyclic fatigue than rotary files. When tested in double curvature canals, reciprocating files also showed higher resistance.

## 1. Introduction

The main objective of root canal therapy is to remove the infected pulp and prevent its reinfection by sealing the root canal space. Chemo-mechanical preparation contributes to the decrease in microorganisms in the root canal in a significant way; however, despite good root canal treatment, microorganisms are capable of surviving in lateral canals and apical branches, inducing endodontic failure [1].

Taking this into account, the introduction of rotary systems represented a revolution by achieving greater cleaning and debridement of the root canal system more effectively and efficiently compared to manual files. The prognosis of root canal treatment has evolved through the development and use of nickel–titanium (NiTi) endodontic rotary instruments, with improvements in mechanical preparation, allowing for more effective cleaning and shaping of the root canal system due to its greater taper and automated motion [2]. The rotary instrumentation technique has showed to be more efficacious in promoting postendodontic healing at short-term review periods compared to the manual instrumentation technique; however, both groups had similar favorable outcomes and survival rates after an extended 5-year monitoring period [3,4,5,6].

Among the multiple advances achieved in recent years, we can highlight the thermal treatment of NiTi alloys, new NiTi alloys [7,8,9,10], the addition of new movements to instrumentation systems [11,12], and innovations in instrument design [13]. Changes in the martensitic phase of the NiTi alloy coupled with the reduced tendency of the file to straighten during use result in a more flexible file [7] with greater resistance to both torsional fracture as a cyclic fatigue [8]. Martensitic transformation of shape memory alloys is a shear-like mechanism which takes place below the transition temperature. Martensitic transformation can be induced by mechanical forces or by temperature changes in a cooling process [10].

Rotating instrument fracture within the canal system remains one of the main concerns and complications during endodontics. The constant evolution of mechanical instrumentation systems, with modifications in the techniques of use, in the design, or in the alloy, make a continuous evaluation of these new files necessary in order to determine the improvements that they contribute with respect to their predecessors [10,11,12].

The fracture of the files Is an unwanted factor that negatively affects root canal treatment that is difficult to solve [13]. Failure of NiTi rotary files can occur due to torsional overload or cyclical bending fatigue [14]. Torsional overload occurs when the tip of the file is blocked within the root canal system, while fatigue is based on a progressive, localized, and permanent structural change that occurs in a material that is subjected to loads with certain repetition patterns, causing the formation of cracks, which in turn give way to total fracture, in the event that it is subjected to a certain number of cycles that exceed its fatigue life limit, and the said stress conditions depend on the characteristics of the material and its use [15]. The polar moment of inertia is a measure of an object’s capacity to oppose or resist torsion when some amount of torque is applied to it on a specified axis; this movement can be considered as the most important cross-sectional factor in determining the torsional resistance of rotary instruments over metal mass and a cross-sectional area [16]. Furthermore, depending on the typology and complexity of the tests, it is possible to obtain different results, in particular regarding static, dynamic tests, or simulated variables of these, with which the literature does not provide clear answers regarding the fracture of the files in endodontics [17,18,19].

To increase the resistance of rotary NiTi files, manufacturers have introduced the use of reciprocating motion. In reciprocating motion, the instrument is first driven in a cutting direction and then rotated in the reverse direction to release it. A 360° rotation is completed with several reciprocating movements, allowing the file to advance towards the apex [20].

The efficiency of reciprocating has been compared to continuous rotation in terms of cutting efficiency and time required to prepare a curved channel. However, despite the studies carried out, no consensus has been reached regarding the advantages of reciprocating movements over continuous movements in relation to their effect on cyclic fatigue [21,22]. Single-file systems have demonstrated the ability to clean and shape the root canal system with fewer instruments, which implies a reduced working time. In addition, they have shown a high capability to maintain the original canal anatomy without removing excess dentin and enhance a more centered preparation compared with rotary multiple-file systems (1); these files have a short learning curve (2). However, single-file systems are subjected to high levels of cyclic and torsional fatigue, which might lead to the fracturing of reciprocating files [9,10,11,12]. The reciprocating movement associated with single-file systems has been shown to extend the lifetime of NiTi rotary files compared with continuous rotation, thus increasing the cyclic fatigue resistance of reciprocating files [12].

Despite the benefits of super elasticity in NiTi alloy, instrument fracture remains a major clinical concern [16,17,18]. Strategies have been implemented to increase the safety and efficiency of NiTi rotary files; this includes the use of new alloys that provide superior mechanical properties and improvements in the manufacturing process [23,24]. These processes carried out during manufacturing have caused the behavior of the NiTi alloy and its mechanical properties to vary according to its thermal/mechanical treatment and its composition [11,25].

The clinical significance of this study is to provide reliable information for the clinician to better choose adequate and safer endodontic instruments in clinical practice. The aim of this study is to know which of the mechanical instrumentation systems currently available, both rotary and reciprocating, has greater resistance to cyclic fatigue, with a null hypothesis (H0) postulating that there would be no difference between reciprocating and rotary endodontic files with regard to cyclic fatigue resistance, as well as an alternative hypothesis (H1) to demonstrate that the use of reciprocating movement has greater resistance to cyclic fatigue.

## 2. Materials and Methods

This systematic review followed the PRISMA (Preferred Reporting Items for Systemic Reviews and Meta-Analyses http://www.prisma-statement.org, accessed on 2 January 2024) guidelines for systematic reviews and meta-analyses [26]. The protocol of this study was recorded in the PROSPERO International Prospective Register of Systematic Reviews (CDR 42021257440). The focused question was given as follows: which mechanical instrumentation system, both rotary and reciprocating, has the highest resistance to cyclic fatigue? Therefore, the PICO question was as follows: Population (P): artificial root canals. Interventions (I): instrumentation with NiTi rotary and reciprocating files. Comparison (C): rotary versus reciprocating files. Outcome (O): cyclic fatigue resistance.

### 2.1. Information Sources and Search Strategy

The search strategy, performed with the Medical Subject Headings terms and key words, was based on the focused population, intervention, control, and outcome research question described earlier. An advanced electronic search was performed in the Medline (via PubMed), Embase, Scopus, SciELO, and WOS databases. Within each concept, we combined the controlled vocabulary (Medical Subject Headings terms) and free key words with the Boolean operators OR and AND. We also used a filter for randomized clinical trials (RCTs) (study design) for the PubMed database (Table 1). The searches were limited to studies published in English from inception to 31 of December 2022. Additional specific searches were performed in the aforementioned databases and were last updated in September 2023.

### 2.2. Eligibility Criteria

Articles were selected for inclusion in this systematic review if they fulfilled all the following criteria: (1) Articles which described in vitro studies that compared cyclic fatigue resistance between rotary and reciprocating instruments on artificial canal models. (2) Articles which assessed both reciprocating and rotary instruments in one study. (3) Articles that compared reciprocating files and rotary files for the kinematics of files and not for other properties such as file alloy, reciprocating range, and so on. (4) Articles that evaluated the main subject of this study regarding reciprocating and rotary files: cyclic fatigue resistance. (5) Articles with a sample size of 20 specimens or more. (6) Articles that use the files according to the manufacturer’s instructions. In addition, articles that compared rotary and reciprocating files for other properties such as file alloy were excluded, articles that failed to meet any of these criteria were excluded, and studies published in languages other than English were not selected. Review articles were not included in the selection.

### 2.3. Assessment of Risk of Bias

The methodological quality of the included studies was judged based on the adaptation of the quality assessment of a previous systematic review conducted considering in vitro studies [27,28,29]. The methodological quality assessment of the included studies was performed independently by the authors (APM, PSL). The following domains were used: (1) randomization of specimens; (2) standardization of samples; (3) standardization of fatigue test devices; (4) materials used according to manufacturer’s instructions; (5) files with similar dimensions; (6) sample size calculation; (7) blinding of fatigue test operator; and (8) correct statistical analysis carried out (Table 2). In cases of disagreement between the examiners, a third examiner was consulted.

The domains reported in the included studies were classified as ‘+’ to register low risk of bias, ‘−‘ to register high risk of bias, and ‘?’ to register unclear parameter. The articles were classified as low risk of bias if 6 or more domains were assigned as low (+), a moderate risk of bias if 4, 5, or 6 domains were assigned as low, and a high risk of bias if only 3 or fewer domains were assigned as low.

### 2.4. Data Analysis

Studies were combined in a meta-analysis using a random effects model by the inverse variance method. The effect size was the mean of the time fracture and cycle fracture. Heterogeneity was assessed using the p value of the Q test for heterogeneity and the I2. The presence of heterogeneity was considered when the *p* value of the Q test was <0.1; moderate when I2 > 50%; and high when I2 > 7 5%. In order to study the sources of heterogeneity, meta-analysis by subgroups was performed with the random effects model according to file movement, curvature of root canal, and curvature angle, and the *p* value of the test for subgroup differences was calculated between groups. Publication bias was analyzed using the trim-and-fill method to adjust for funnel plot asymmetry. Statistical analysis was performed with R softw version 4.3.2 are and meta package.

## 3. Results

### 3.1. Study Selection and Data Collection Process

**Figure 1:**
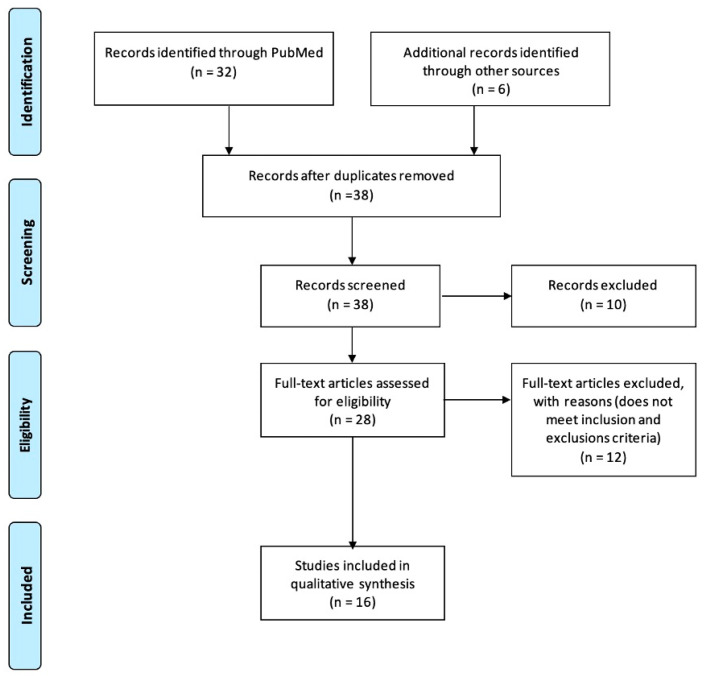
Flow diagram showing the process of identifying, screening, and reasons for excluding the studies.

After database screening, we removed duplicates and selected possible eligible articles according to title and abstracts, obtained full-text articles, and classified them according to the inclusion criteria (Figure 1).

The characteristics of the included studies are summarized in Table 3. A total of 16 studies were included in the meta-analysis. It was necessary to carry out the analysis in two different groups depending on whether the cyclic fatigue resistance was measured in “time to fracture” (TTF) or “number of cycles to fracture” (NCF). To carry out the analysis, the mean and standard deviation (SD) of the “time to fracture” (TTF) and “number of cycles to fracture” (NCF) of the files systems in each study were extracted.

### 3.2. Time to Fracture (TTF)

#### 3.2.1. Overall Meta-Analysis

**Figure 2:**
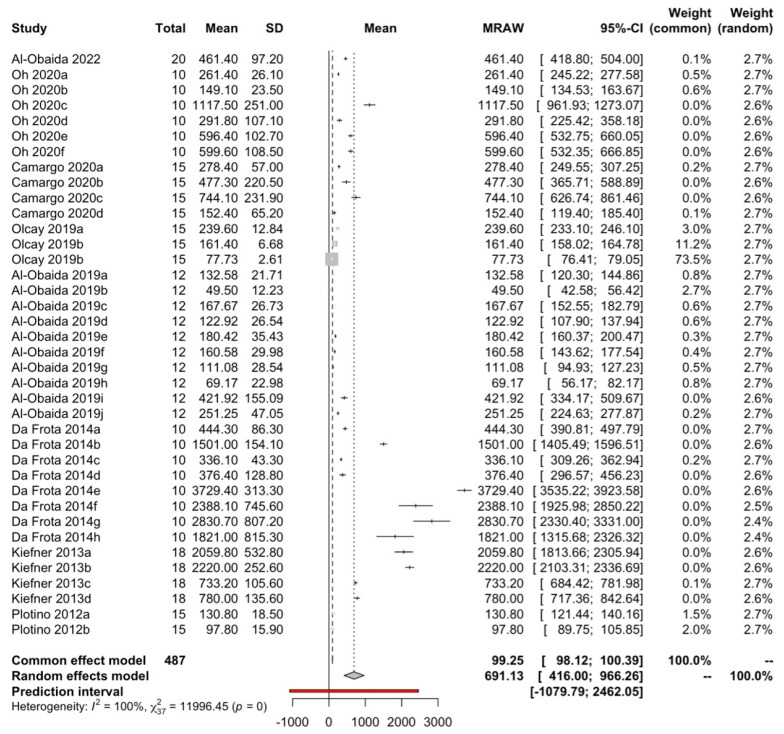
Forest plot of cyclic fatigue resistance measured as time to fracture [30,31,34,35,36,39,41,43].

Using a random effects model with the inverse variance method, 38 means from 8 studies with a total of 487 observations have been combined. A mean fracture time of 691.1 s with a 95% confidence interval between 416.0 and 966.3 has been estimated (Figure 2). The heterogeneity of the meta-analysis was maximum with I2 = 100%, Q test = 11,996.5; *p* < 0.001. 

#### 3.2.2. Subgroup Analysis

To study the possible sources of heterogeneity, subgroup analysis was performed.

##### File Movement

**Figure 3:**
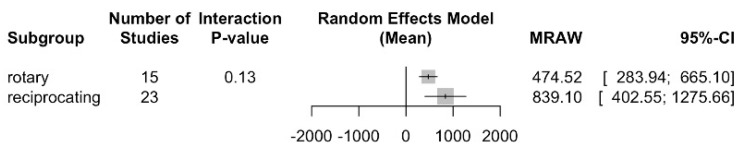
Time to fracture overall analysis between rotary and reciprocating files.

Regarding file movement, a TTF for rotary files was determined in 474.5 s (95% IC between 283.9 and 665.1) with an I2 = 99.7%, and 839.1 for reciprocating (IC 95% between 402.5 and 1275.7) with an I2 = 99.6%. There were no statistically significant differences between the subgroups with a Q test = 2.25; *p* value = 0.134 (Figure 3).

##### Curvature of Root Canal

**Figure 4:**
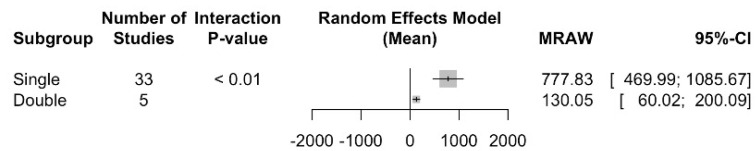
Time to fracture subgroup analysis according to curvature of the in vitro root canals.

A TTF for single curvature root canals of 777.8 s (CI 95% between 470 and 1085.7) and an I2 = 99.7% was determined, and 130.1 s was determined for double curvature canals (CI 95% between 60 and 200.1) with an I2 = 98.9%. There were statistically significant differences between the subgroups with a Q test = 16.2; *p* value < 0.001 (Figure 4).

##### Curvature Angle

**Figure 5:**
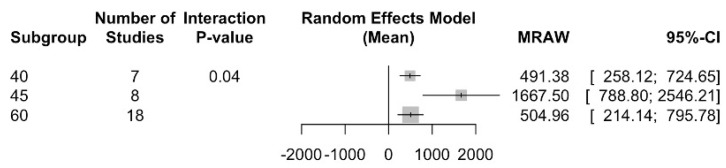
Time to fracture subgroup analysis according to curvature angle.

For a 40º curvature angle, a TTF of 491.4 s (95% CI between 258.1 and 724.6) and an I2 = 99% were determined; for a 45º curvature angle, 1667.5 s (CI 95% between 788.8 and 2546.2) and an I2 = 99.6 were achieved; and, finally, for a 60º curvature angle, 505 s (CI 95% between 214.1 and 795.8) and an I2 = 99.8% were determined. There are statistically significant differences between the subgroups with a Q test = 6.54; *p* value < 0.037 (Figure 5).

### 3.3. Number of Cycles to Fracture (NCF)

#### 3.3.1. Overall Meta-Analysis

**Figure 6:**
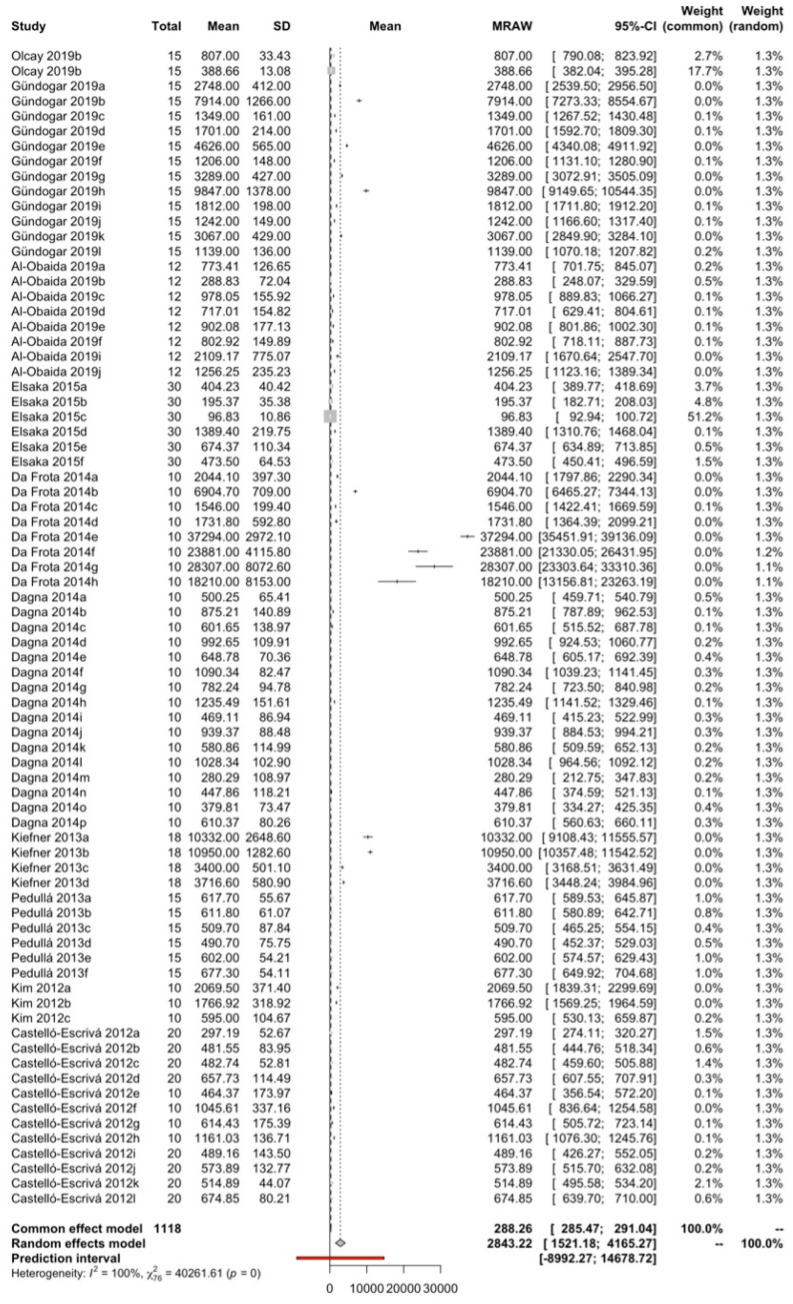
Cyclic fatigue resistance measured as number of cycles to fracture (NCF) [11,31,32,34,35,37,38,40,41,42].

Using a random effects model with the inverse variance method, 77 means from 10 studies with a total of 1118 observations have been combined. The mean number of cycles to fracture was estimated at 2842.2, with a 95% confidence interval between 1521.2 and 4165.3. The heterogeneity of the meta-analysis was maximum with an I2 = 99.8%, Q test = 40,261.6; *p* < 0.001 (Figure 6).

#### 3.3.2. Subgroup Analysis

To study the possible sources of heterogeneity, subgroup analysis was performed.

##### File Movement

**Figure 7:**
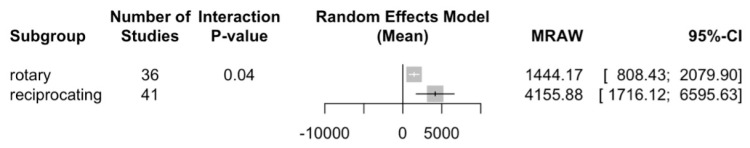
Number of cycles to fracture overall analysis between rotary and reciprocating files.

Regarding file movement, an NCF for rotary systems was determined in 1444.2 (CI 95% between 808.4 and 2079.9) with an I2 = 99.9%, and for reciprocating file systems, 4155.9 (CI 95% between 1716.1 and 6595.6) and an I2 = 99.5% were determined. There are statistically significant differences between the subgroups with a Q test = 4.44; *p* value = 0.035, making reciprocating files more resistant to cyclic fatigue than rotary ones (Figure 7).

##### Curvature of Root Canal

**Figure 8:**
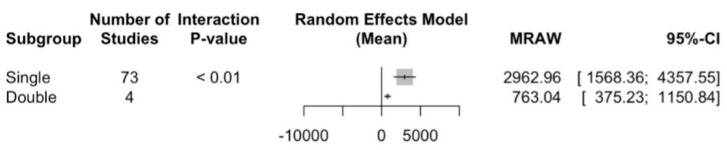
Number of cycles to fracture subgroup analysis according to curvature of the in vitro root canal.

Regarding curvature, an NCF of 2963 for the single curvature (CI 95% between 1568.4 and 4357.6) and an I2 = 99.8% have been determined, and for double curvature of the root canal, 763 (CI 95% between 375.2 and 1150.8) and an I2 = 99% were determined. There are statistically significant differences between the subgroups with a Q test = 8.87; *p* value = 0.003 (Figure 8).

##### Curvature Angle

**Figure 9:**
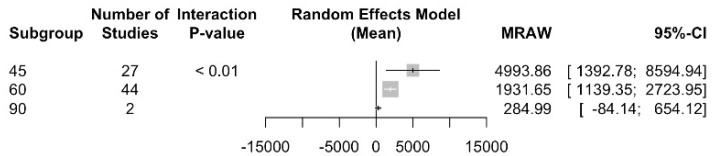
Number of cycles to fracture subgroup analysis according to angle of curvature.

Regarding the root canal curvature angle, an NCF for the 45º curvature angle was determined at 4993.47 (CI 95% between 1392.8 and 8594.9) with an I2 = 99.6%; for the 60º curvature angle model, 1931.7 (CI 95% between 1139.4 and 2724) and an I2 = 99.7 were determined; and, finally, for the 90º curvature angle, 285 (CI 95% between 0 and 654.1) and an I2 = 99.9% were achieved. There are statistically significant differences between the subgroups with a Q test = 19.4; *p* value < 0.001 (Figure 9).

Analysis of these studies demonstrated that there are no statistically significant differences in cyclic fatigue resistance between rotary files and reciprocating files when this variable is measured as time to fracture. Nevertheless, when it is measured as the number of cycles to fracture, we found statistically significant differences (*p* = 0.03), making reciprocating files more resistant to cyclic fatigue than rotary ones (Figure 7 and Figure 8).

## 4. Discussion

The fracture of NiTi instruments can result from two situations: torsional fatigue and cyclic fatigue [34,35,36]. The present systematic review and meta-analysis was carried out to compare the cyclic fatigue resistance of rotary and reciprocating file systems. One of the limitations of this research was comparing cyclic fatigue fracture resistance for motion, excluding the influence of parameters like alloy or design of the file. It is known that the influence on the cyclic fatigue resistance of the file is more significant in some cases from factors like alloy and shape, rather than motion of the file itself. To conduct a meta-analysis, it is essential that different studies on files have similar conditions. Some studies have compared the motion while keeping conditions like file design or heat treatment consistent. The influence of the alloy and design on cyclic fatigue resistance and its implications for the findings of our study is of utmost importance; however, we have chosen to eliminate these factors and focus only on the movement, without incorporating the rest of the variables.

After data extraction, the results were divided according to the measurement of the cyclic fatigue resistance, that is, in terms of time to fracture and number of cycles to fracture of the files. This has been considered a limitation of the present study and highlights the need for an international standard for testing the cyclic fatigue resistance of NiTi endodontic files. Several self-designed devices and methods have been used with different results [21,44]. However, none of these custom-made devices have been capable of dynamically testing the cyclic fatigue of NiTi endodontic files in vitro with an automatic detection system and an anatomically based artificial root canal. Finding a standardization system that reflects the characteristics of the tooth would be a great advance to be able to understand and compare resistance to the cyclic fatigue of endodontic files.

Reciprocation and rotary instrumentation provide different root canal morphologies; there are several studies that demonstrate that the middle third of root canals is a critical region where remnants are packed and spread in the buccal–lingual sides of canals. ESEM-EDX detected a fine layer of filling remnants in all root thirds, suggesting a larger canal contamination than the X-rays and CBCT examinations revealed [45,46].

Another limitation to highlight is that, in the data processing, we have not used the new tool to analyze the risk of bias (RoB2) [47]. The main reason for this is that according to Minozzi et al. [48], RoB 2 is a detailed and comprehensive tool but difficult and demanding, even for raters with substantial expertise in systematic reviews. Calibration exercises and intensive training are needed before its application to improve reliability.

### 4.1. Cyclic Fatigue Resistance Measured as Time to Fracture (TTF)

Design of the file dimensions, angle of curvature of the root canal, experience of the operator, torque, and speed of rotation are factors that influence the cyclic fatigue resistance of rotary instruments [35,36]. Cyclic fatigue is the cause of 94.4% of fractured instruments among postgraduate students and is more frequent in larger sizes [49]. Several studies have shown that fracture time is also related to the speed of instrument rotation: instruments are more resilient when operating at lower speeds [34,50]. The Reciproc system (RCP, VDW, Munich, Germany) rotates in clockwise and counterclockwise directions at a speed of approximately 300 rpm, and the WaveOne system (WO, Dentsply Maillefer, Ballaigues, Switzerland) rotates at a rotational speed of 350 rpm [51,52]. The rotational speed during the reciprocating motion is not constant, unlike what occurs with continuous rotary systems, as the electric motor has certain mechanical limitations in converting rotation direction [37]. Thus, acceleration and deceleration in both rotation directions generate less tension on the instrument and therefore provide greater cyclic fatigue resistance [30,34,36,41,43], as observed in the present study.

The influence of file design on cyclic fatigue resistance is controversial. Some authors suggest that the file cross-section has a major impact on its half-life and strength [40]. It is therefore important to note that the single use of reciprocating files reduces the risk of accumulating fatigue in the metal [31].

### 4.2. Cyclic Fatigue Resistance Measured as Number of Cycles to Fracture (NCF)

Cyclic fatigue is the degeneration process that occurs in endodontic files when they are subjected to cyclical loads within the tooth root canal; this can influence the origin and spread of the type of fracture that may occur. The number of cycles performed is related to the intensity of the tension generated by the compression and traction forces in the curved part of the instrument during instrumentation and is cumulative [53]. To calculate the number of cycles to fracture, multiply the time (in seconds) to failure by the number of rotations, regardless of the direction of rotation [38].

The results obtained in the present study partly rejected the null hypothesis which stated that there would be no differences in cyclic fatigue resistance between rotary and reciprocating endodontic files. Regarding file movement, the present meta-analysis found that reciprocating files have better cyclic fatigue resistance than rotary files, with a statistically significant difference when it is measured in NCF.

In reciprocating motion, the instrument is first driven in a cutting direction and then rotated in the reverse direction to release it. A 360° rotation is completed with several reciprocating movements, allowing the file to advance towards the apex [54]. The use of reciprocating motion has been shown to increase fatigue resistance compared to continuous rotation and therefore extend the life of the NiTi instrument [55]. The results of this study confirm that reciprocating file systems are more resistant to cyclic fatigue than rotary ones.

Kim et al. [37], after evaluating the resistance to cyclic fatigue and torsional resistance of two reciprocating motion systems, Reciproc (R25) and WO file, reported greater resistance of both files compared with Protaper^®^ F2 (Dentsply Maillefer, Ballaigues, Switzerland) in a continuous rotation. Their results are consistent with those obtained by Dagna et al. [42] when comparing R25 and WO with OneShape^®^ (Micro Mega, Besançon, France) and PTF2.

Kiefner et al. [35] compared the cyclic fatigue resistance of R25 and R40 with MTwo 25 (VDW, Munich, Germany) and Mtwo 40 and found that the reciprocating movement of the NiTi instruments increases the instrument cyclic fatigue resistance. Other authors have found similar results [11,31,32,38,56].

Reciproc and WaveOne systems are instruments designed to cut in a counterclockwise direction. The greatest rotation, which corresponds to the cutting direction, occurs counterclockwise, causing dentin removal and advancing the instrument in the canal. The clockwise angle of rotation is less, thus allowing it to be unlocked and moved safely through the root canal, reducing the risk of instrument fracture. These angles of rotation are determined with respect to the torsion properties of each instrument and are specific to each system [36,40,57]. Therefore, from its design, the aim of the reciprocating movement is to decrease the risk of torsion fracture since the angle of rotation in the counterclockwise direction was designed to be smaller than the elastic limit of the instrument.

The main risk factor with respect to failure due to cyclic fatigue seems to be the curvature of the canal due to the multiple stresses that occur while the files shape the root canals, involving the NiTi core of the files in stresses of flexion and cyclic fatigue [58,59,60]. Therefore, in canals with large curvatures, little can be done to prevent or reduce these stresses on the files except to apply our knowledge of endodontics with the glyde path, preflaring, etc. [61,62].

Elsaka et al. [32] reported that NCF was significantly affected by the type of instrument and angle of curvature (*p* < 0.001). These authors found a significant interaction between the type of instrument and angle of curvature (*p* < 0.001). Wave One^®^ Gold had a significantly higher resistance to cyclic fatigue than the OneShape^®^ instrument (MicroMega, Besançon, France) in all groups (*p* < 0.05). The results showed that the 45° angle of curvature yielded the highest NCF, while the 90° angle of curvature generated the least NCF in the two systems tested.

Al-Obaida et al. [31] found that Reciproc^®^ Blue (VDW, Munich, Germany) had a significantly higher mean NCF, followed by WaveOne Gold, Reciproc, and WaveOne in this study. In the artificial canal with double curvature, Reciproc Blue had a significantly higher mean NCF, followed by Reciproc, WaveOne Gold, and WaveOne. No significant difference between WaveOne Gold and both Reciproc groups was found, but all three were significantly greater in cyclic fatigue resistance than the WaveOne group.

“Cycles to fracture” is used to measure the cyclic fatigue resistance of continuous rotary file systems. However, due to the unique characteristics of reciprocation files, it is challenging to precisely calculate these cycles. Therefore, “time to fracture” is being used instead for reciprocation files. In this meta-analysis, there were authors who compared NCF [10,11,32,34,37,38,40,42] and others who compared TTF [30,33,36,39,43], or even both in the same study [31,35,41], which is detailed in the results.

We have not found statistically significant differences in cyclic fatigue resistance between rotary files and reciprocating files when this variable is measured as “time to fracture”, while when it is measured as “number of cycles to fracture”, a significant difference is observed; however, the values achieved are much higher in reciprocating movement (474.5 s for rotary and 839.1 s for reciprocating). Furthermore, regarding curvature and angle of curvature, we find significant differences.

Channel angles was a variable incorporated and studied by the authors reviewed. It would be more appropriate to include descriptions related to the impact of specific movements for files at certain angles, emphasizing which movement has a more significant effect at particular angles and even adding new movements in reciprocation [21,63], which opens the way for new studies.

The present study presents a direct application to the clinical setting since reciprocating systems provided higher cyclic fatigue resistance when the number of cycles to fracture is measured. Clinicians should choose reciprocating motion systems to reduce the probability of file fractures, particularly in root canal systems with a pronounced angle and/or curvature radius. However, it is very difficult to eliminate other factors that influence resistance to cyclic fatigue in addition to kinematics.

## 5. Conclusions

This meta-analysis showed that reciprocating files have better resistance to cyclic fatigue than rotary files. When tested in double curvature canals, reciprocating files also showed higher resistance than rotary files.

## Figures and Tables

**Table 1:** Search strategy.

Search Details	Results
(“rotary files” [Title/Abstract] OR “rotary instruments” [Title/Abstract] OR “rotary” [Title/Abstract]) AND (“reciprocating files”[Title/Abstract] OR “reciprocating instruments” [Title/Abstract] OR “reciprocating” [Title/Abstract]) AND (“cyclic fatigue resistance” [Title/Abstract] OR “stress resistance”[Title/Abstract] OR “stress fracture” [Title/Abstract])	32
(“rotary files” [Title/Abstract] OR “rotary instruments” [Title/Abstract] OR “rotary” [Title/Abstract] OR (“reciprocating files”[Title/Abstract] OR “reciprocating instruments” [Title/Abstract] OR “reciprocating” [Title/Abstract])) AND (“cyclic fatigue resistance” [Title/Abstract] OR “stress resistance”[Title/Abstract] OR “stress fracture” [Title/Abstract])	244
(“rotary files” [Title/Abstract] OR “rotary instruments” [Title/Abstract] OR “rotary” [Title/Abstract]) AND (“reciprocating files” [Title/Abstract] OR “reciprocating instruments” [Title/Abstract] OR “reciprocating” [Title/Abstract])	380
“rotary files” [Title/Abstract] OR “rotary instruments” [Title/Abstract] OR “rotary” [Title/Abstract]	11,204
“reciprocating files” [Title/Abstract] OR “reciprocating instruments” [Title/Abstract] OR “reciprocating” [Title/Abstract]	2836
“cyclic fatigue resistance” [Title/Abstract] OR “stress resistance” [Title/Abstract] OR “stress fracture” [Title/Abstract]	9421

**Table 2:** Risk of bias assessment of the included studies.

	Randomization	Standardization	Standardization of fatigue test devices	Manufacturers instructions	Files dimensions	Sample size calculation	Blinding of operator	Statistical analysis	Risk of bias
Al-Obaida et al., 2022 [30]	−	+	+	+	+	+	−	+	LOW
Hou et al., 2020 [10]	−	+	+	+	+	+	−	+	LOW
Al-Obaida et al., 2019 [31]	−	+	+	+	+	+	−	+	LOW
Elsaka et al., 2015 [32]	−	+	+	+	+	+	−	+	LOW
Karataşlıoglu et al., 2016 [33]	−	+	+	+	+	+	−	+	LOW
Da Frota et al., 2014 [34]	−	+	+	+	+	−	−	+	MEDIUM
Kiefner et al., 2014 [35]	−	+	+	+	+	+	−	+	LOW
Plotino et al., 2012 [36]	−	+	+	+	+	+	−	+	LOW
Kim et al., 2012 [37]	−	+	+	+	+	+	−	+	LOW
Castelló-Escrivá et al., 2012 [38]	−	+	+	+	+	+	−	+	LOW
Gündogar et al., 2019 [11]	−	+	+	+	+	−	−	+	MEDIUM
Ribeiro-Camargo et al., 2020 [39]	−	+	+	+	+	−	−	+	MEDIUM
Pedullà E et al., 2013 [40]	−	+	+	+	+	−	−	+	MEDIUM
Olcay et al., 2019 [41]	−	+	+	+	+	+	−	+	LOW
Dagna et al., 2014 [42]	−	+	+	+	+	+	−	+	LOW
Oh et al., 2020 [43]	−	+	+	+	+	−	−	+	MEDIUM

**Table 3:** Characteristics of the studies included in the systematic review and meta-analysis.

Author, Year	Sample Size	File System, Diameter/ Taper	Speed/Torque	Fatigue Test Device	Curvature Angle (Θ) Radius ®	Model Setup	Outcome	Results		Statistical Significance
Al-Obaida et al., 2022 [30]	n = 20 per group	WO 25/08 RP 25/08 PTF2 25/08 UNICONE 25/08	WO/RP: Reciprocating movement (300 rpm/2 Ncm) UNICONE: Reciprocating movement (300 rpm/3.1 Ncm) PTF2: 150–300 rpm/1.5–3 Ncm	Stainless steel artificial canals inserted in methacrylate	Θ = 40° r = 5 mm	Static	Time to fracture (TTF) (seconds)	WO = 15.37 RECIPROC = 11.88 UNICONE = 4.7 PTF2= 7.69		WO instrument had the highest cyclic fatigue resistance among the tested groups (*p* < 0.05), while Unicone had the lowest cyclic fatigue resistance.
Hou et al., 2020 [10]	n = 20 per group	PTG 25/08 HEDM 25/08 RPB 25/08 WOG 25/07	RP/WOG: Reciprocating movement PTG: 300 rpm/3.1 Ncm HEDM: 500 rpm/2.5 Ncm	Stainless steel artificial canals inserted in methacrylate	Θ = 60° r = 3 mm	Static Dynamic	Number of cycles to fracture (NFC)	Static: PTG = 267.67 HEDM = 723 RPB = 1024.5 WOG = 483.46		The dynamic cyclic fatigue resistance test showed significantly higher NCF than the static cyclic fatigue resistance test in the PTG and EDM (*p* < 0.05). There was no significant difference between the RPB and WOG (*p* > 0.05).
								Dynamic: PTG = 904.8 HEDM = 2692.71 RPB = 1087.42 WOG = 712.05		
Oh et al., 2020 [43]	n = 40 per group.	WOG 25/07 RPB 25/08 HEDM 25/08	WOG/RPB: Reciprocating HEDM: 500rpm	Stainless steel artificial canals inserted in methacrylate	Θ = 40° r = 5 mm	Static	Time to fracture (seconds)	22 °C (RT) WOG = 261.4 RPB = 1117.5 HEDM = 596.4		At RT, RPB demonstrated the longest fracture time, followed by HDM, and WOG (*p* < 0.05). At BT, HDM had the longest fracture time, followed by RPB, and WOG (*p* < 0.05).
								37 °C (BT) WOG = 149.1 RPB = 291.8 HEDM = 599.6		
Ribeiro-Camargo et al., 2020 [39]	n = 15 per group	HYFCM 25/06 GEN 25/04 WOG 25/07 PTU F2 25/08	HYFCM: 500 rpm 2.5N/CM GEN: reciprocating (90°/30°) WOG reciprocating PTU: 300 rpm 3.0 N/CM	Stainless steel artificial canals inserted in methacrylate	Θ = 60° r = 3 mm	Static	Time to fracture (seconds)	HYFCM = 744.1 GEN= 477.3 WOG= 278.4 PTU = 152.4		HYFCM and GEN showed the best results for cyclic fatigue, torsional failure, and flexural resistance, followed by WOG and PTU (*p* < 0.05).
Al-Obaida et al., 2019 [31]	n = 24 per group	WO 25.08 WOG 25.07 RP 25.08 RPB 25.08 TFA 25.06	WO/WOG: Reciprocating (350 rpm) RP/RPB: Reciprocating (300 rpm)	Stainless steel artificial canals inserted in methacrylate	Single curvature: Θ = 60º r = 5 mm	Static	Time to fracture (seconds) Number of cycles to fracture (NFC)	Single curvature:		RECIPROC BLUE files exhibited significantly greater cyclic fatigue resistance compared with other files tested in an S-shaped artificial canal.
								TTF: WO = 132.58 WOG = 167.67 RP = 180.42 RPB = 421.92 TFA = 111.08	NCF: WO = 773.41 WOG = 978.05 RP = 902.08 RPB = 2109.17	
					Double curvature: Θ = 60º r = 5 mm + Θ = 70º r = 2 mm			Double curvature		
								TTF WO = 49.50 WOG = 122.92 RP = 160.58 RPB = 251.25 TFA = 69.17	NCF WO = 288.83 WOG = 717.01 RP = 802.92 RPB = 1256.25	
Olcay et al., 2019 [41]	N = 15 per group	WOG 25.07 PTN 26.06 TS 25.06	WOG:350 rpmPTN:300 rpm TS: 300 rpm	Stainless steel artificial canals inserted in methacrylate	Θ- 60º R- 5 mm	Static	Time to fracture (seconds) Number of cycles to fracture (NFC)	TTF: WOG = 239.60 PTN = 161.40 TS = 77.73 NCF: PTN = 807 TS = 388.66		WOG > PTN > TS according to TTF results (*p* = 0.05). PTN > TS according to NCF results (*p* = 0.05).
Gündogar et al., 2019 [11]	N= 45 per group	RPB 25.08 HEDM 25.08 WOG 25.07 TFA 25.08	RPB/WOG: Reciprocating HEDM: 500 rpm 2.5 N/cm TF: TF Adaptative	Stainless steel artificial canals inserted in methacrylate	Θ- 60º R- 5mm	Static	Number of cycles to fracture (NFC)	Air 20 °C TF= 1242 WOG = 1701 HEDM = 3289 RPB = 2748 Water 20 °C TF = 3067 WOG = 4626 HEDM = 9847 RPB = 7914 Water 35 °C TF = 1139 WOG = 1206 HEDM = 1812 RPB = 1349		RPB exhibited the best cyclic fatigue resistance in 20 °C air and distilled water environments (*p* < 0.05). There was no significant difference in the cyclic fatigue resistance of the files in a 35 °C water environment (*p* > 0.05).
Elsaka et al., 2015 [32]	N = 90 per group	OS 25/.06 WOG 25/.08	OS: 400 rpm, 4 N/cm WOG: reciprocating	Stainless steel artificial canals inserted in methacrylate	Θ- 45º, 60º, 90º R- 5 mm	Static	Number of cycles to fracture (NFC)	OS: 45° = 404.23 60° = 195.37 90° = 96.83 WOG: 45° = 1389.4 60° = 674.37 90° = 473.5		WOG instrument had a higher cyclic fatigue resistance than OS (*p* < 0.05).
Karataşlıoglu et al., 2016 [33]	N = 20 per group	OS 25/.06 WOG 25/.08	G1: 150º-30º G2: 210–30º G3: 360º-30º G4: Continuous rotation (350 rpm 2.5 N/cm)	Stainless steel artificial canals inserted in methacrylate	Θ- 60º R- 3 mm	Static	Time to fracture (seconds)	G1: 150º-30º OS 104.9 WOG 36.9 G2: 210–30º OS 177.8 WOG 84.2 G3: 360º-30º OS 221.8 WOG 88.0 G4: Continous rotation OS 313.0 WOG 99.4		All the reciprocating motions resulted in extended fatigue life when compared with continuous rotation (*p* < 0.05).
Da Frota et al., 2014 [34]	N= 20 per group	PTU 25/.08 WO 25/.08 Mtwo 25/.06 RP 25/.08	RP/WO: Reciprocating PTU: 280 rpm, 2.3 N/cm. Mtwo: 280 rpm, 2.3 N/cm	Stainless steel artificial canals inserted in methacrylate	Θ- 45º R- 5mm	Static	Number of cycles to fracture (NFC)	No axial displacement: PTU = 6904.7 Mtwo = 1731.8 RP = 2388.1 WO = 18,210.0 Axial displacement: PTU = 2044.1 Mtwo = 1546.0 RP = 37,294.0 WO = 28,307.0		Cyclic fatigue resistance was greater for reciprocating systems than for continuous rotary systems, irrespective of axial displacement (*p* < 0.05).
Dagna et al., 2014 [42]	N = 40 per group	OS 25.06 RP 25.08 WO 25.08 PTF2 25/08	OS:350 rpm 4 N/cm RP/WO: Reciprocating PT F2: 300 rpm 2 N/cm	Stainless steel artificial canals inserted in methacrylate	Canal 1: Θ- 60º R- 8 mm Canal 2: Θ- 45º R- 8 mm Canal 3: Θ- 60º R- 5 mm Canal 4: Θ- 45º R- 5 mm	Static	Number of cycles to fracture (NFC)	Canal 1: OS = 500.27 RP = 648.78 WO = 469.11 PTF2 = 280.29 Canal 2: OS = 875.21 RP = 1090.34 WO = 939.37 PTF2 = 447.86 Canal 3: OS = 601.65 RP = 782.24 PTF2 = 379.81 Canal 4: OS = 992.65 RP = 1235.49 WO = 1028.34 PTF2 = 610.37		RP showed the highest cyclic fatigue resistance. OneShape and WO files showed similar cyclic fatigue resistance values, higher than PTF2.
Kiefner et al., 2014 [35]	N = 18 per group.	RP 25.08 RP 40.06 Mtwo 25.06 Mtwo 40.05	R25 in reciprocation movement; R40 in reciprocation movement; M25 in rotary movement; M40 in rotary movement;	Stainless steel artificial canals inserted in methacrylate	Θ- 60º R- 5mm	Dynamic	Time to fracture (seconds) Number of cycles to fracture (NFC)	TTF: R25 = 2059.8 R40 = 2220 M25 = 733.2 M40 = 780 NCF: R25 = 10,332 R40 = 10,950 M25 = 3400 M40 = 3716.6		Reciprocating movement increased the cyclic fatigue resistance of NiTi instruments (*p* < 0.05).
Pedullà et al., 2013 [40]	N = 15 per group	RP 25.08 WO 25.08 Mtwo 25.06 TF 25.06	RP/WO: reciprocating Mtwo/TF: 300 rpm	Stainless steel artificial canals inserted in methacrylate	Θ- 60º R- 5 mm	Static	Number of cycles to fracture (NFC)	RP25 = 617.70 WO = 509.70 M25 = 602 TF = 677.30		The cyclic fatigue resistance of the 2 reciprocating motions was significantly higher than the continuous rotation in each brand (*p* < 0.001).
Plotino et al., 2012 [36]	N = 15 per group	RP 25/.08 WO 25/.08	RP/WO: reciprocating	Stainless steel artificial canals inserted in methacrylate	Θ- 60º R- 5 mm	Static	Time to fracture (seconds)	RP= 130.8 WO= 97.8		Reciprocating instruments were associated with a significantly higher cyclic fatigue resistance than WaveOne instruments.
Kim et al., 2012 [37]	N = 10 per group.	RP 25.08 WO 25.08 PTF2 25.08	RP/WO: reciprocating PTF2: 300 rpm	Stainless steel artificial canals inserted in methacrylate	Θ- 45° R- 6 mm	Static	Number of cycles to fracture (NFC)	RP = 2069.50 WO = 1766.92 PTF2 = 595.00		Reciprocating files seem to have superior mechanical properties.
Castelló-Escrivá et al., 2012 [38]	N = 184	PTF2 25/.08 WO 25/.08 TF 25/.08	300 rpm	Stainless steel artificial canals inserted in methacrylate	Canal 1: Θ- 60° R- 8 mm Canal 2: Θ- 45° R- 8 mm Canal 3: Θ- 60° R-5 mm Canal 4: Θ- 45° R- 5 mm	Static	Number of cycles to fracture (NFC)	Canal 1: PTF2 = 297.19 WO = 464.37 TF = 489.16 Canal 2: PTF2 = 481.55 WO = 1045.61 TF = 573.89 Canal 3: PTF2 = 482.74 WO = 614.43 TF = 514.89 Canal 4: PTF2 = 657.73 WO = 1161.03 TF = 674.85		Reciprocating movement of WO showed a longer cyclic fatigue life than conventional rotary movement of TF and ProTaper.

## Data Availability

The datasets used and/or analyzed during the current study are available from the corresponding author on reasonable request.
